# Does logging and forest conversion to oil palm agriculture alter functional diversity in a biodiversity hotspot?

**DOI:** 10.1111/acv.12074

**Published:** 2013-10-09

**Authors:** F A Edwards, D P Edwards, T H Larsen, W W Hsu, S Benedick, A Chung, C Vun Khen, D S Wilcove, K C Hamer

**Affiliations:** 1 School of Biology, University of LeedsLeeds, UK; 2 Department of Animal and Plant Sciences, University of SheffieldSheffield, UK; 3 School of Marine and Tropical Biology, James Cook UniversityCairns, QLD, Australia; 4 Science and Knowledge Division, Conservation InternationalArlington, VA, USA; 5 Department of Ecology, Evolution, and Environmental Biology, Columbia UniversityNew York, NY, USA; 6 School of Sustainable Agriculture, Universiti MalaysiaKota Kinabalu, Sabah, Malaysia; 7 Sepilok Forest Research Centre, Sabah Forestry DepartmentSandakan, Sabah, Malaysia; 8 Woodrow Wilson School and Department of Ecology and Evolutionary Biology, Princeton UniversityPrinceton, NJ, USA

**Keywords:** Borneo, deforestation, ecosystem services, habitat change, palm oil, tropical forest

## Abstract

Forests in Southeast Asia are rapidly being logged and converted to oil palm. These changes in land-use are known to affect species diversity but consequences for the functional diversity of species assemblages are poorly understood. Environmental filtering of species with similar traits could lead to disproportionate reductions in trait diversity in degraded habitats. Here, we focus on dung beetles, which play a key role in ecosystem processes such as nutrient recycling and seed dispersal. We use morphological and behavioural traits to calculate a variety of functional diversity measures across a gradient of disturbance from primary forest through intensively logged forest to oil palm. Logging caused significant shifts in community composition but had very little effect on functional diversity, even after a repeated timber harvest. These data provide evidence for functional redundancy of dung beetles within primary forest and emphasize the high value of logged forests as refugia for biodiversity. In contrast, conversion of forest to oil palm greatly reduced taxonomic and functional diversity, with a marked decrease in the abundance of nocturnal foragers, a higher proportion of species with small body sizes and the complete loss of telecoprid species (dung-rollers), all indicating a decrease in the functional capacity of dung beetles within plantations. These changes also highlight the vulnerability of community functioning within logged forests in the event of further environmental degradation.

## Introduction

Land-use change is the major driver of ecosystem degradation and biodiversity loss globally (Nepstad *et al.*, 1999; Brooks *et al.*, 2002; Nelson *et al.*, 2006; Laurance, 2007), with an ever-growing proportion of the world's natural habitats being altered by anthropogenic activities (Morris, 2010). Roughly 13 million hectares of forest were converted annually between 2000 and 2010, concentrated within the tropics and principally for agricultural expansion (Hansen *et al.*, 2008; FAO, 2010). In addition, 403 million hectares of tropical forest are designated for logging (Blaser, Sarre & Johnson, 2011), with the rate of logging about 20 times that of forest clearance (Asner *et al.*, 2009).

The impacts of land-use change on biodiversity are often examined, particularly in tropical ecosystems, using measures of diversity (e.g. species richness and Simpson or Shannon diversity indices) that take no account of differences in species' life-history traits and ecological niches. Yet changes in environmental conditions following disturbance may well act as a filter, allowing only a narrow spectrum of traits to persist (Hamer *et al.*, 2003; Gray *et al.*, 2007; Cardinale *et al.*, 2012; Fauset *et al.*, 2012). As a consequence, such traditional diversity measures may be inappropriate indicators of changes in community structure, underestimating the true extent of biodiversity loss following disturbance (Cardinale *et al.*, 2012; Mouillot *et al.*, 2013). One solution is to use measures of functional diversity, which seek to quantify the range of functional (i.e. trait) differences among species in a community (Tilman, 2001; Petchey & Gaston, 2006), thus bridging the gap between species diversity and species composition, and giving insight into potential resilience and recovery of species in response to land-use change (Koh, Sodhi & Brook, 2004; Hillebrand, Bennett & Cadotte, 2008; Ockinger *et al.*, 2010; Mouillot *et al.*, 2013).

Despite the value of functional diversity metrics in inferring ecosystem processes (de Bello *et al.*, 2010; Naeem, Duffy & Zavaleta, 2012; Mouillot *et al.*, 2013), the impacts of tropical land-use change on functional diversity are poorly understood. Examination of the literature identified just 12 studies that quantified the functional diversity of tropical communities across a terrestrial disturbance gradient (Table 1). Of these studies, only three compared the functional diversity of communities in logged forest with those in primary forest. They found that amphibian functional diversity was higher in primary forest (Ernst, Linsenmair & Rödel, 2006), but that arboreal and avian functional diversity were not different (Baraloto *et al.*, 2012; Edwards *et al.*, 2013).

**Table 1:** Studies investigating functional diversity in the tropics across a land-use gradient

Taxa	Geographic region	Land-use change	Functional metric(s)	Study
Amphibians	Ivory Coast and Central Guyana	Primary and selectively logged forest	FD	Ernst *et al.*, 2006
Ants	Brazilian Atlantic forest	Forest fragmentation (size)	FEve	Leal *et al.*, 2012
Ants	Brazilian Atlantic forest	Secondary forest (age)	FD, FAD	Bihn, Gebauer & Brandl, 2010
Birds	Malaysian Borneo	Primary and selectively logged forest, and oil palm	FD, FEve, FDiv	Edwards *et al.*, 2013
Birds	Brazilian Amazon	Unburned and burned (frequency) forest	MPD, MNTD	Hidasi-Neto, Barlow & Cianciaruso, 2012
Birds, Plants, Mammals	Costa Rica to USA	Temperate and tropical, natural, semi-natural and agricultural habitats	FD	Flynn *et al.*, 2009*
Dung Beetles	Mexico	Forest fragmentation (size)	FRic, FEve, FDiv	Barragán *et al*., 2011
Dung Beetles	Mexico	Continuous forest, forest fragmentation and pasture	FRic, FEve, FDiv	Barragán *et al*., 2011.
Trees	French Guiana	Primary and selectively logged forest gaps	FRic, FEve, FDiv	Baraloto *et al.*, 2012
Trees	Mexico	Secondary forest (age)	FD	Lohbeck *et al.*, 2012
Understory plants	Solomon Islands	Primary forest, secondary forest, plantations and pastures	FRic, FEve, FDis	Katovai, Burley & Mayfield, 2012
Utilitarian plants	Madagascar	Continuous and fragmented forest (varying degradation), and agricultural habitats	FD	Brown *et al.*, 2013
Woody plants	Brazilian Cerrado	Fire (frequency)	FD	Cianciaruso *et al.*, 2012

Functional metric abbreviations: FAD, functional attribute diversity; FD, functional dendrogram; FDis, functional dispersion; FDiv, functional divergence; FEve, functional evenness; FRic, functional richness; FSpe, functional specialization; MNTD, mean nearest taxon distance; MPD, mean pairwise distance. Superscript (^*^) represents a meta-analysis.

In addition, only one previous study has investigated the impacts of oil palm agriculture on functional diversity (Table 1; Edwards *et al.*, 2013), yet this is a widespread and rapidly expanding crop globally (Fitzherbert *et al.*, 2008; Gibbs *et al.*, 2010). In Southeast Asia, the conversion of forest (both primary and logged) to oil palm agriculture has been rampant, with several million hectares of oil palm plantation replacing forest over the last two decades (Koh & Wilcove, 2008; Gibbs *et al.*, 2010; Reynolds *et al.*, 2011). Dung beetles provide key ecosystem functions and services, including nutrient recycling, soil aeration, secondary seed dispersal and parasite suppression (Nichols *et al.*, 2008), as their habit of breaking apart dung piles and distributing the material away from the source. However, only one previous study has examined impacts of land-use change on the functional diversity of dung beetles (in Mexico; Barragán *et al*., 2011), yet these are globally widespread, highly abundant (Hanski & Cambefort, 1991), sensitive to environmental changes (Larsen, Williams & Kremen, 2005; Nichols *et al.*, 2007) and key indicators for other taxonomic groups, especially mammals (Nichols *et al.*, 2009).

In this study, we address these key knowledge gaps by investigating the impacts of land-use change on the taxonomic and functional diversity of dung beetles in the global biodiversity hotspot of Sundaland, Southeast Asia (Myers *et al.*, 2000). We examine a gradient of increasing habitat disturbance from primary forest through once-logged and twice-logged forest to oil palm. We test the hypothesis that disturbance acts as an environmental filter, selecting species more functionally similar than expected by chance and hence leading to lowered functional diversity in disturbed habitats.

## Materials and methods

### Study location

Our study is based within the Yayasan Sabah (YS) logging concession and adjacent oil palm plantations, in eastern Sabah, Malaysian Borneo (4°58′ N, 117°48′ E). Within the YS concession is 45 200 ha of primary forest in the Danum Valley Conservation Area and Palum Tambun Watershed Reserve, which is dominated numerically by valuable timber species of the family Dipterocarpaceae (Fisher *et al.*, 2011). Adjacent to this primary forest is the 238 000 ha Ulu Segama-Malua Forest Reserve (US-MFR) containing selectively logged forest, of which 97 000 ha (41%) has undergone a single rotation of timber extraction (once-logged forest) and the remaining area has undergone two rotations of logging (twice-logged forest). The first rotation of timber extraction took place between 1987 and 1991, with commercial stems > 0.6 m diameter removed to yield ≈ 115 m^3^ of timber per ha (Fisher *et al.*, 2011). Twice-logged locations were relogged between 2001 and 2007 with the minimum harvested tree diameter reduced to > 0.4 m, yielding an additional 15–72 m^3^ of timber per ha (Edwards *et al.*, 2011; Fisher *et al.*, 2011). Surrounding the US-MFR are oil palm plantations, with sampling locations restricted to mature plantations (10–15 years old), with density of ≈ 100 palms ha^−1^ (Edwards *et al.*, 2010).

### Dung beetle sampling

Fieldwork was conducted between August and October 2009, and between February and September 2011. In each of our four habitats, we created four sampling sites that were widely spaced across the landscape. Sites within a habitat were separated by ≥ 2 km, and distances between sites in different habitats ranged from 1 to 92 km.

We used standardized baited pitfall traps to sample dung beetles (Coleoptera: Scarabaeidae: Scarabaeinae) across the four habitat types. Within each site, we created two transects (separated by 500–900 m), and along each transect we placed five pitfall traps baited with human dung at 100 m intervals (total traps = 160; see Edwards *et al.*, 2011 for further details), which was sufficient to ensure independence (Larsen & Forsyth, 2005). We left traps for 4 days and rebaited after 48 h, with beetles collected every 24 h (Edwards *et al.*, 2011). We used reference collections (T. Larsen) housed at the Forest Research Centre, Sandakan, Malaysia and Smithsonian Museum, Washington DC, USA for species determinations.

### Data analysis

#### Species richness, diversity, evenness and composition

We compared dung-beetle species richness between forested habitats and oil palm using sample-based rarefaction curves with 95% confidence intervals and standardized by the total abundance of individuals in a particular habitat (Gotelli & Colwell, 2001). To assess the accuracy of our sampling, we calculated the average of four commonly used abundance based estimators of species richness (ACE, CHAO1, JACK1 and Bootstrap) using ESTIMATES v. 8.2 (University of Connecticut, Storrs, CT, USA). We measured species diversity using the Shannon-Wiener index and calculated species evenness using Pielou's evenness index in Vegan package (Oksanen *et al.*, 2011).

To investigate the change in species composition between habitats, we used a non-metric multidimensional scaling ordination (Clarke & Warwick, 2001), using the isoMDS function with Bray-Curtis dissimilarity measure within the MASS package (Magurran, 2004). Communities were standardized as a proportion of the total number of individuals on each transect. To test for significant differences in composition, we used a permutational multivariate analysis of variance (ADONIS function in Vegan; Oksanen *et al.*, 2011) with 1000 permutations.

#### Measuring functional diversity

We examined five traits: behavioural guild, diel activity, body size, diet breath and diet preference (Supporting Information Table S1). Behavioural guilds were categorized into three main groups: rollers (telecoprid), tunnellers (paracoprid) and dwellers (endocoprid) (for descriptions see Hanski & Cambefort, 1991). Information on species behavioural guilds and diel activity (diurnal or nocturnal foragers) were obtained from the literature (Davis, 1999; Krikken & Huijbregts, 2007; Slade *et al.*, 2007; Qie *et al.*, 2011; Slade, Mann & Lewis, 2011) and personal observations. We used a dial calliper to measure body length (pygidium to anterior margin of pronotum) and elytra width to the nearest 0.01 mm (*n* = 1–27 individuals per species). Body size was then calculated as the product of these two variables (Larsen, Lopera & Forsyth, 2008). We investigated diet breadth with alternative baited traps: rotting vertebrate carrion (*n* = 19 trap days), rotting fruit (*n* = 18 trap days) or rotting fungus (*n* = 16 trap days). Trap design was identical as for traps baited with dung, beetles were collected every 24 h but traps were left for 48 h. Following Larsen *et al*. (2008), we used the number of different baits a species was attracted to (range = 1–4) as a measure of dietary breadth, and the bait a species was most frequently recorded on as a measure of dietary preference, standardized by the number of trap days (abundance/number trap days) (Supporting Information Table S2). Functional traits were not highly correlated (Kendall correlation: *τ* < 0.54).

Having obtained trait data, we used the formulae of Villéger, Mason & Mouillot (2008, Villéger *et al*., 2010, Villéger, Novack-Gottshall & Mouillot, 2011) to calculate five complementary measures of functional diversity: (1) functional richness (FRic), which quantifies the volume of functional space that a set of species occupies; (2) functional evenness (FEve), which describes how species' abundances are distributed throughout the occupied functional space; (3) functional divergence (FDiv), which summarizes the variation in species abundances with respect to the centre of functional space; (4) functional specialization (FSpe), which describes how functionally unique a community is relative to the regional pool of species, and; (5) functional dissimilarity (FDis), which indicates the overlap of functional space between two or more communities. In these methods, traits act as coordinates in functional space, thus identifying a species' functional niche (Villéger *et al*., 2008). Traits were given equal weighting and species were weighted by their relative abundance. Because our functional traits were a mixture of variable types, we calculated a distance matrix using the Gower distance measure, before running a principal coordinates analysis (PCoA) to calculate a new trait matrix of transformed coordinates (Villéger *et al*., 2008). Four PCoA axes were used to calculate the functional measures using a multidimensional convex hull to position species in functional trait space.

FSpe was measured as the average distance of a set of species from the centre of functional space, relative to the regional pool of all species (Villéger *et al*., 2010). FDis was measured as the volume of functional space that two communities share (Villéger *et al*., 2011). When two communities overlap completely, FDis is equal to zero, and as the overlap in functional space is reduced so dissimilarity increases towards one.

#### Observed and expected functional diversity

To assess whether disturbance leads to the selection of more functionally similar species than expected by chance, we compared the standardized effect size (SES) of our four functional diversity metrics (FRic, FEve, FDiv and FSpe) across habitats. We defined SES as *[(observed – mean expected)/standard deviation of expected]*. Expected functional diversity metrics were calculated from 1000 random communities generated from the overall regional species pool. An independent swap algorithm was used to maintain species richness and species frequency occurrence in the random communities (picante package of R) (Gotelli, 2000; Kembel *et al.*, 2010). We then used one-sample t-tests with *μ* = 0 to determine whether the SES of each functional diversity metric was significantly different from zero.

#### Comparing among habitats

To check that our results were independent of spatial scale (Hamer & Hill, 2000), each of our species and functional diversity measures were calculated and compared at a large scale (the overall habitat) and a small scale (individual transects). For the latter, we used linear mixed-effect models (LME), including site as a random effect to account for repeated measures. Species abundance was square-root transformed prior to analysis. We also used a Monte-Carlo permutation test for Moran's I statistic (moran.mc within the spdep package), using our model residuals with 1000 repetitions, to test whether or not our transect level results were influenced by spatial autocorrelation. All analyses were run in R v.2.13.2 (R Development Core Team, 2011).

## Results

### Species richness, diversity and composition

**Figure 1:**
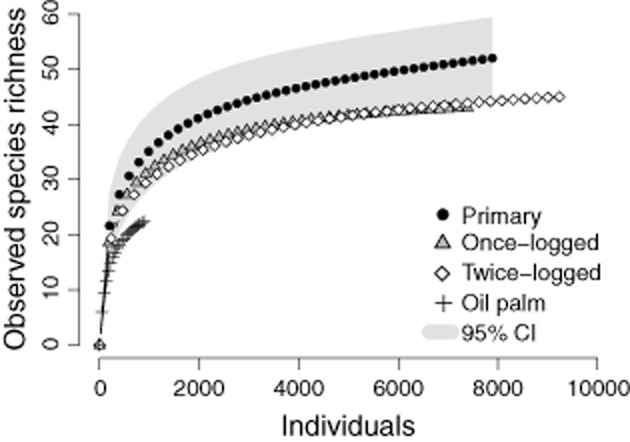
Observed species richness, calculated from sample-based rarefaction curves and scaled to show the number of individuals on the *x*-axis for dung beetles across a disturbance gradient. Grey shading represents 95% confidence interval (CI) of primary forest.

Across the four habitats, we recorded 26 285 individual dung beetles of 65 species. The four common estimators of species richness suggest that we sampled ≥ 89% of species in each of the four habitats (Table 2). There was a decrease in the overall species richness, diversity, evenness and abundance of individuals in oil palm compared to forest, both at the habitat scale (Fig. 1; Table 2) and on individual transects (Table 2; LME: species richness, *F*_3,12_ = 18.39, *P* < 0.001; abundance *F*_3,12_ = 12.51, *P* < 0.001; species diversity *F*_3,12_ = 16.14, *P* < 0.001; evenness *F*_3,12_ = 5.99, *P* = 0.01). In contrast, logged forest communities did not differ significantly from those in primary forest with respect to species richness, diversity, evenness or abundance (all *P* ≥ 0.1) (Table 2).

**Table 2:** Summary of taxonomic species metrics in primary forest, once-logged forest, twice-logged forest and oil palm plantations

Measure	Primary	Once-logged	Twice-logged	Oil palm
Habitat level:				
Abundance	7885	7386	9231	1783
Sobs^c^	52	43	45	25
Sest^d^	58	45	48	27
Sobs/Sest^e^	0.89	0.96	0.93	0.93
Species diversity^f^	2.75	2.67	2.50	1.85
Species evenness^g^	0.69	0.71	0.66	0.58
Transect level:				
Sobs	32 ± 1.10^a^	27 ± 2.50^a^	29 ± 1.00^a^	12 ± 1.30^b^
Species diversity	2.62 ± 0.08^a^	2.39 ± 0.13^a^	2.37 ± 0.05^a^	1.36 ± 0.13^b^
Species evenness	0.76 ± 0.02^a^	0.73 ± 0.26^a^	0.71 ± 0.25^a^	0.57 ± 0.20^b^

Means (±1se) are at the transect level. Superscripts (^a,b^) represent pairwise differences tested at *P* ≤ 0.05.

c Observed species richness.

d Estimated species richness.

e Proportion of species recorded.

f Measured using Shannon diversity index.

g Measured using Pielou's index.

**Figure 2:**
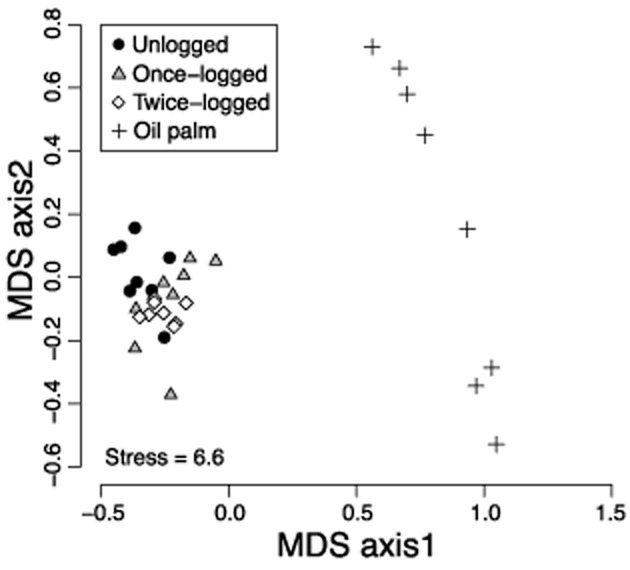
Non-metric multidimensional scaling (MDS) ordination of community assemblages between unlogged (primary) forest, once-logged forest, twice-logged forest and oil palm at the transect scale.

Species composition differed significantly between forest and oil palm (Fig. 2; ADONIS: *r*^2^ = 0.54, d.f. = 3, *P* = 0.0001), with the three most abundant species in each forest habitat (*Paragymnopleurus sparsus*, *Sisyphus thoracicus* and *Onthophagus cervicapra*) replaced in oil palm by three congeneric species (*O.* sp. *B, O. obscurior, O. rugicollis*). Additionally, 37 of 52 species recorded in primary forest (71%) did not occur in samples from oil palm, while a further nine species occurred in oil palm but not in forest. The species assemblage of primary forest was significantly different from that of both once-logged (*r*^2^ = 0.20, d.f. = 1, *P* = 0.001) and twice-logged forest (*r*^2^ = 0.20, d.f. = 1, *P* = 0.02), but the assemblages in the two-logged forest treatments did not differ (*r*^2^ = 0.08, d.f. = 1, *P* = 0.29).

### Functional diversity

Functional richness, divergence and evenness did not differ among the three forest treatments (Table 3; all *P* > 0.16). FSpe was significantly higher in primary forest than in once-logged forest [LME (value ± se): 0.31 ± 0.12, d.f. = 12, *P* = 0.03; overall model F_3,12_ = 50.11, *P* < 0.001] but not in twice-logged forest (Table 3). However, all forest treatments were more functionally specialized than expected from random community assemblages (Supporting Information Fig. S2: all *P* < 0.01). FDis was high between forest and oil palm (> 98% non-overlap), but was low among all three of the forest treatments (< 13% non-overlap) (Supporting Information Fig. S1).

**Table 3:** Habitat and transect (mean ± 1se) scale functional diversity indices in primary forest, once-logged forest, twice-logged forest and oil palm plantations. FRic, FEve and FDiv are bounded between 0 and 1, and FRic was standardized by a theoretical community of all 65 species in the regional pool

Functional measure	Primary	Once-logged	Twice-logged	Oil palm
Habitat level:				
FRic^d^	1.00	0.87	0.99	0.01
FEve^e^	0.28	0.31	0.29	0.45
FDiv^f^	0.74	0.68	0.72	0.54
FSpe^g^	2.17	1.85	2.06	0.87
Transect level:				
FRic	0.58 ± 0.07^a^	0.42 ± 0.07^a^	0.48 ± 0.09^a^	0.35 ± 0.09^b^
FEve	0.42 ± 0.03	0.39 ± 0.02	0.39 ± 0.02	0.37 ± 0.04
FDiv	0.76 ± 0.03^a^	0.69 ± 0.03^a^^b^	0.73 ± 0.02^a^	0.58 ± 0.05^b^
FSpe	2.21 ± 0.08^a^	1.90 ± 0.10^b^	2.07 ± 0.04^a^^b^	0.87 ± 0.01^c^

Superscripts (^a,b,c^) represent pairwise differences tested at *P* ≤ 0.05.

d Functional richness.

e Functional evenness.

f Functional divergence.

g Functional specialization.

**Figure 3:**
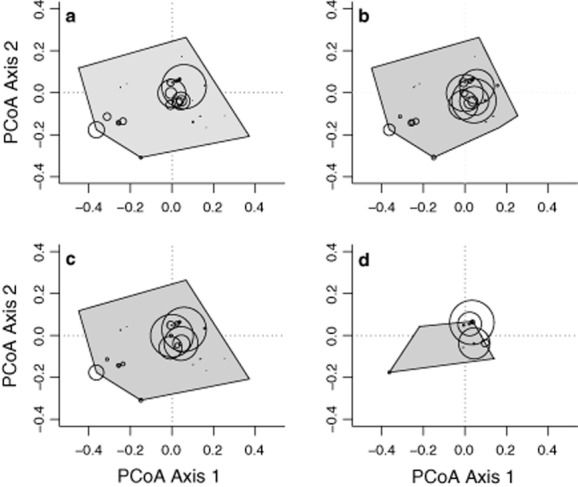
Functional richness of dung beetle communities, described as the minimum convex hull enclosing all species of a community and represented as the volume of functional space filled (denoted here by the area of the grey polygon), in (a) primary forest, (b) once-logged forest, (c) twice-logged forest and (d) oil palm. The black circles are proportional to the relative abundance of species in an individual habitat. Functional richness was much lower in oil palm than elsewhere.

Functional richness, divergence and specialization were all much lower in oil palm than in any of the three forest habitats, at both spatial scales (Table 3; LME: FRic, *F_3,12_* = 11.52, *P* < 0.001; FDiv, *F_3,12_* = 3.68, *P* = 0.004; FSpe, *F*_3,12_ = 50.11, *P* < 0.001). Observed FRic (one-sample t-test: t_7_ = −7.90, *P* < 0.01) and FSpe (t_7_ = −11.85, *P* < 0.01) were also significantly lower than expected from the regional species pool in oil palm (Supporting Information Fig. S2a,d). FEve, however, was not significantly different in oil palm than elsewhere (Table 3; *F_3,12_* = 0.37, *P* = 0.8). The functional space occupied by dung beetles in oil palm showed major constrictions (Fig. 3), indicating a marked reduction or complete loss of some functional traits. In particular, telecoprid species (dung-rollers) were abundant in forest but absent from oil palm, the proportion of nocturnal species was lower in oil palm (8%) than in forest (primary = 25%, once-logged = 30%, twice-logged = 22%), and the three most abundant species were smaller in oil palm (body size, mean ± se: 20.83 ± 3.98 mm) than elsewhere (44.97 ± 26.02 mm). There was no spatial autocorrelation across transects for model residuals of any of the functional diversity metrics (Moran's I test: *P* ≥ 0.2 in each case).

## Discussion

Primary rainforests in Southeast Asia are highly threatened by rampant logging and the expansion of large-scale oil palm agriculture (Sodhi *et al.*, 2010; Wilcove *et al.*, 2013), yet this study is among the first assessments of how land-use change affects functional diversity in the region. We found marked reductions in the taxonomic and functional diversity of dung beetles following the conversion of forest to oil palm. In contrast, however, there was very little evidence of any such loss within logged forests, despite significant changes in species composition in comparison to primary forest and even after repeated rotations of logging. These data provide evidence for functional redundancy of dung beetles within primary forest, as also suggested for birds in Amazonian forests (Hidasi-Neto, Barlow & Cianciaruso, 2012). Our results also emphasize the importance of degraded forests as refugia for biodiversity (Edwards *et al.*, 2011; Woodcock *et al.*, 2011), and highlight the potential consequences of biodiversity losses for the support of ecosystem processes within agricultural systems.

Dung beetle communities in oil palm are compositionally distinct from those of primary and logged forest (Table 2), with a shift of numerically dominant species, a loss of numerous forest specialists and the addition of a much smaller number of new, presumably disturbance-tolerant species (Figs 2 and 3). These findings support previous work from western Africa that recorded lower species richness and diversity of dung beetles in oil palm plantations compared to logged and primary forests (Davis & Philips, 2005). However, we found significantly lower abundance of dung beetles in oil palm than in forest, whereas the opposite was found in Africa (Davis & Philips, 2005). This variability highlights the need for a more geographically complete understanding of the impacts of oil palm as it expands across tropical regions and replaces both forest and natural grasslands (Koh *et al.*, 2011; Garcia-Ulloa *et al.*, 2012). Assessing the ability of species to persist within remnant forest patches and disperse across the wider land-use matrix will also be critical to evaluating strategies to enhance biodiversity within agricultural landscapes (Edwards *et al.*, 2010).

The dramatic decline that we observed in FRic following conversion of forest to oil palm indicates that the loss of forest species (Fig. 3) was not counterbalanced by the addition of new, disturbance-tolerant species that could either fill vacant functional niches or occupy different functional roles (Table 3; Fig. 3, Supporting Information Figs S1 and S2a,d). The community changes in oil palm show strong evidence for environmental filtering (SES < 0,Fig. S2a,b). In particular, the absence of rollers within oil palm may have been due to altered microclimatic conditions including increased soil temperatures (Lucey & Hill, 2012) decreasing the survival of roller larvae, which typically occur at shallower depths within the soil (Sowig, 1995; Larsen, 2012). We also found a higher proportion of small-bodied species in oil palm, possibly because maximum temperatures in this habitat come closer to exceeding the thermoregulatory tolerance of larger-bodied species, again leading to reduced survival (Nichols *et al.*, 2013). In addition, many dietary generalists (feeding on ≥3 bait types) and species feeding on dung plus carrion were absent from oil palm (Supporting Information Table S2), in contrast to previous work indicating that species with broader diets were less vulnerable to local extinctions (Qie *et al.*, 2011).

Our results suggest that the transition from primary or logged forest to oil palm results in such environmental stresses, particularly due to microclimatic changes, that large subsets of forest species are driven to local extinction irrespective of their dietary breadth or specialization. The absence of rollers within oil palm is particularly important in functional terms, given that they are highly abundant in forests and are behaviourally distinct from tunnellers and dwellers, moving nutrients and seeds away from concentrated dung piles and burying dung balls at shallower depths. In addition, dung removal rate, tunnel depth and volume of dung buried are all positively related to body size, and so the smaller species occurring within oil palm are likely to bury less dung and at lower depths (Slade *et al.*, 2007; Nichols *et al.*, 2008). Changes in the diversity and abundance of nocturnal versus diurnal species may also lead to longer exposure of dung at the surface, resulting in higher gaseous losses of nitrogen (Yamada *et al.*, 2007). Consequently, our results suggest that the functional ability of dung beetles in oil palm is likely to be compromised.

The much lower taxonomic and functional diversity of dung beetles in oil palm also highlights the potential losses that could arise from further degradation of logged forests, for instance through wildfires, which can also act as strong environmental filters and alter microclimatic conditions within the forest (Peres, Barlow & Haugaasen, 2003; Slik & Van Balen, 2006; Lindenmayer *et al.*, 2009; Brodie, Post & Laurance, 2012). Measuring additional functional traits could help in predicting the longer-term impacts of logging and forest conversion. For instance, measures of endothermy and fecundity could aid our understanding of the impacts of microclimatic changes and the likelihood of extinction lags caused by disturbance.

In conclusion, we provide new data on the impacts of land-use change on tropical dung beetles. Contrary to our expectations, even repeated timber harvests did not simplify the functional structure of dung beetle assemblages in Bornean rainforests, despite significant changes in species composition, highlighting the importance of protecting these degraded, logged-over forests. However, conversion of forests to oil palm greatly reduced both species and functional diversity. We suggest ecosystem functioning will be negatively impacted in oil palm, but quantifying the precise consequences across all habitats remains a major knowledge gap. For instance, the retention of forest patches and riparian strips within oil palm estates could support ecosystem services such as nutrient recycling within plantations, but data are needed to address this issue. Our results support previous findings that traditional metrics such as species richness and composition can hide important information about the impacts of land-use change on species traits and functional ecology. The two approaches provide different but complementary mechanisms for understanding human impacts on biodiversity, which can contribute to future conservation and agricultural management decisions (Loyola *et al.*, 2008; Vandewalle *et al.*, 2010; Hidasi-Neto *et al.*, 2012).

## Supporting Information

Figure S1 Functional dissimilarity measured as the overlap of species within functional space. Species are plotted within four-dimensional functional trait space. (a) Axes 1 and 2: primary and twice-logged forest (light grey), once-logged forest (mid-grey) and oil palm (dark grey), and (b) Axes 3 and 4: primary, once-logged and twice-logged forest (light grey); oil palm (dark grey). **Figure S2.** The mean standardized effect size (SES) of functional diversity metrics: (a) functional richness; (b) functional evenness; (c) functional divergence; (d) functional specialization in each habitat. *SES* = *[(Observed − mean Expected)/**sd* *Expected]*. Expected functional metrics are calculated from 1000 randomizations of the regional pool of species in which species frequency occurrence and species richness are maintained. SES > zero indicates greater functional diversity than the regional species pool. **Table S1.** Broad trait categories. Scale indicates the type of trait, functional trait shows how the trait is measured and functional importance suggests the impacts of the trait for ecosystem functioning. **Table S2.** Abundance of species in each habitat, abbreviations: primary forest (P), once-logged forest (1L), twice-logged forest (2L) and oil palm plantation (OP), the functional traits used: the abundance of species visiting dung, carrion, fruit and fungi bait types, the average body size (measured to the nearest mm), the guild and the diel activity of species.
