# Supplementary material for: Does logging and forest conversion to oil palm agriculture alter functional diversity in a biodiversity hotspot?
Source: Anim Conserv. 2013 Oct 9;17(2):163–73. doi: 10.1111/acv.12074 (PMC4372061; doi:10.1111/acv.12074)
Supplement: Figure S1 — Functional dissimilarity measured as the overlap of species within functional space. Species are plotted within four-dimensional functional trait space. (a) Axes 1 and 2: primary and twice-logged forest (light grey), once-logged forest (mid-grey) and oil palm (dark grey), and (b) Axes 3 and 4: primary, once-logged and twice-logged forest (light grey); oil palm (dark grey). Figure S2. The mean standardized effect size (SES) of functional diversity metrics: (a) functional richness; (b) functional evenness; (c) functional divergence; (d) functional specialization in each habitat. SES = [(Observed − mean Expected)/sd Expected]. Expected functional metrics are calculated from 1000 randomizations of the regional pool of species in which species frequency occurrence and species richness are maintained. SES > zero indicates greater functional diversity than the regional species pool. Table S1. Broad trait categories. Scale indicates the type of trait, functional trait shows how the trait is measured and functional importance suggests the impacts of the trait for ecosystem functioning. Table S2. Abundance of species in each habitat, abbreviations: primary forest (P), once-logged forest (1L), twice-logged forest (2L) and oil palm plantation (OP), the functional traits used: the abundance of species visiting dung, carrion, fruit and fungi bait types, the average body size (measured to the nearest mm), the guild and the diel activity of species. [file acv0017-0163-SD1.doc]

**Supplementary Material**

**Does logging and forest conversion to oil palm agriculture alter functional diversity in a biodiversity hotspot?**

Felicity A. Edwards^1*^, David P. Edwards^2^, Trond H. Larsen^3^, Wayne W. Hsu^4^, Suzan Benedick^5^, Arthur Chung^6^, Chey Vun Khen^6^, David S. Wilcove^7^, Keith C. Hamer^1^

^1^ *School of Biology, University of Leeds, Leeds, LS2 9JT, UK.*

^2^ *Centre for Tropical Environmental and Sustainability Science (TESS) and School of Marine and Tropical Biology, James Cook University, Cairns, Queensland 4878, Australia.*

^3^ *Science and Knowledge Division, Conservation International, 2011 Crystal Drive, Suite 500, Arlington, Virginia 22202 USA*

^4^ *Department of Ecology, Evolution, and Environmental Biology, Columbia University, New York 10027 USA*

^5^ *School of Sustainable Agriculture, Universiti Malaysia Sabah, Malaysia*

*^6^ Sepilok Forest Research Centre, Sabah Forestry Department, Sandakan, Sabah, Malaysia*

^7^ *Woodrow Wilson School and Department of Ecology and Evolutionary Biology, Princeton University, Princeton, NJ 08544, USA*

** Address correspondence to F.A. Edwards, email* [*bs08f2a@leeds.ac.uk*](mailto:bs08f2a@leeds.ac.uk)

Contents:

Table S1: The functional relevance of traits

Table S2: The abundance of species using different baited traps

Figure S1: A graphical representation of functional dissimilarity

Figure S2: The mean standardized effect size (SES) of functional diversity metrics

Table S1: Broad trait categories. Scale indicates the type of trait, Functional Trait shows how the trait is measured, and Functional Importance suggests the impacts of the trait for ecosystem functioning.

Table S2: Abundance of species in each habitat, abbreviations: primary forest (P), once-logged forest (1L), twice-logged forest (2L) and oil palm plantation (OP), the functional traits used: the abundance of species visiting dung, carrion, fruit and fungi bait types, the average body size (measured to the nearest mm), the guild and the diel activity of species.

Figure S1:

Figure S1: Functional dissimilarity measured as the overlap of species within functional space. Species are plotted within four-dimensional functional trait space. **(a)** Axes 1 and 2: primary and twice-logged forest (light grey), once-logged forest (mid-grey), and oil palm (dark grey), and **(b)** Axes 3 and 4: primary, once-logged, and twice-logged forest (light grey); oil palm (dark grey).


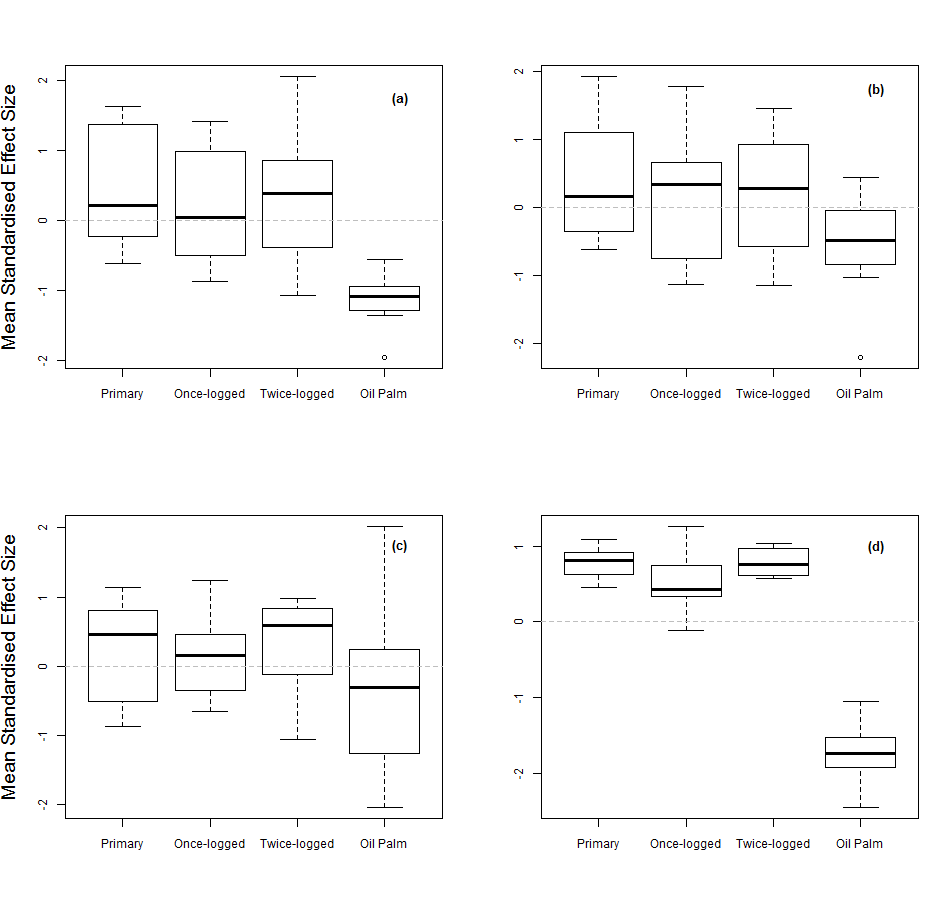
Figure S2:

Figure S2: The mean standardized effect size (SES) of functional diversity metrics: **(a)** functional richness; **(b)** functional evenness; **(c)** functional divergence; **(d)** functional specialisation in each habitat. *SES = ([Observed – mean Expected]/SD Expected)*. Expected functional metrics are calculated from 1000 randomisations of the regional pool of species in which species frequency occurrence and species richness are maintained. SES > zero indicates greater functional diversity than the regional species pool.
